# Borneol and Α-asarone as adjuvant agents for improving blood–brain barrier permeability of puerarin and tetramethylpyrazine by activating adenosine receptors

**DOI:** 10.1080/10717544.2018.1516005

**Published:** 2018-10-19

**Authors:** Jun-Yong Wu, Yong-Jiang Li, Le Yang, Yi-Yun Hu, Xiong-Bin Hu, Tian-Tian Tang, Jie-Min Wang, Xin-Yi Liu, Da-Xiong Xiang

**Affiliations:** a Department of Pharmacy, The Second Xiangya Hospital, Central South University, Changsha, Hunan, China;; b Institute of Clinical Pharmacy, Central South University, Changsha, Hunan, China;; c Hunan Provincial Engineering Research Center of Translational Medicine and Innovative Drug, Changsha, Hunan Province, China

**Keywords:** Borneol, α-asarone, puerarin, tetramethylpyrazine, blood–brain barrier, adenosine receptor

## Abstract

Puerarin (PUE) and tetramethylpyrazine (TMP) are central nervous system (CNS) drugs used in cerebrovascular diseases. Poor brain–blood barrier (BBB) permeability limited their clinical application. Borneol and α-asarone have been proposed as an oral brain-targeting enhancer. In this study, we aimed to first evaluate the ‘orifice-opening’ effect of borneol and α-asarone, both aromatic resuscitation drugs, on improvement of brain delivery of PUE and TMP and second to investigate whether the enhancing effects were associated with adenosine receptors (ARs)-mediated trans-BBB pathway. *In vitro* BBB model was established and borneol and α-asarone significantly increased the cumulative amount of permeated PUE and TMP and the enhancing effects could be counteracted by AR inhibitors. Borneol and α-asarone could decrease expression of ZO-1, an important BBB junction protein, but inversely increase the expression of A_1_AR and A_2A_AR. *In vivo* pharmacokinetic study also confirmed that oral co-administration of borneol or α-asarone significantly increased AUC_brain_ for PUE and TMP. These results suggested that borneol and α-asarone are both effective adjuvant agents for delivery of PUE and TMP to the brain.

## 1. Introduction

The blood–brain barrier (BBB) refers to the network of microvasculature of the vertebrate central nervous system (CNS) (Abbott et al., 2010). BBB selectively blocks uncontrolled transcellular passage of substances between the circulation and CNS and also restricts paracellular diffusion of solutes by a network of intercellular junctional complexes that tightly connect the endothelial cells (Huber et al., 2001). BBB is of great significance for maintaining the stability of CNS but also prevents the distribution of drugs to the brain (Garcia-Garcia et al., 2005). Therefore, the clinical application of CNS drugs is limited by their poor brain bioavailability.

Puerarin (PUE) is the primary bioactive ingredient from the root of *Pueraria lobata (willd.) ohwi*. PUE has therapeutical effects on cerebral infraction for its various pharmacological activities on cerebrovascular system, such as anti-inflammation (Wei et al., 2014; Zhang et al., 2014), anti-oxidation (Wang et al., 2014a; Zhong et al., 2014), antiplatelet aggregation (Liu et al., 2006), anti-apoptosis (Li et al., 2018), vasodilation (Wang et al., 2014c) and improving cerebral microcirculation (Wu et al., 2014; Yuan et al., 2014). Tetramethylpyrazine (TMP) is the major pharmacological ingredient of traditional Chinese medicine Chuanxiong Hort (*Rhizoma Chuanxiong*), TMP has been used as one of the most effective treatment for ischemic stroke (Sun et al., 2008). TMP also protects the brain from cerebral injury by anti-platelet (Sheu et al., 2000). However, the clinical application of PUE and TMP are limited by their poor brain bioavailability due to low BBB permeability.

Studies have reported that adenosine receptors (ARs)-mediated trans-BBB is an important pathway for drug delivery to the CNS (Carman et al., 2011; Kim & Bynoe, 2015). Of note, A_1_AR and A_2A_AR are important neuromodulators of the CNS. Under physiological conditions, adenosine, which is synthesized by CD73 (extracellular-5′-nucleotidase), activates A_1_AR and A_2A_AR and reduces the expression of tight junction protein ZO-1 and increases paracellular gap, which facilitate drug delivery to CNS (Carman & Springer, 2008; Fredholm et al., 2011).

Borneol and α-asarone, both aromatic resuscitation drugs, have been considered as effective ‘orifice-opening’ agents (Wang et al., 2014b; Zhang et al., 2017). However, the mechanism for their mechanism of improving drug delivery to the brain, such as tight junction opening, remains to be further elucidated (Cai et al., 2014).

Therefore, this study aims to investigate whether borneol and α-asarone as aromatic ‘orifice-opening’ agents improved blood–brain barrier permeability of PUE and TMP and the underlying mechanism associated with A1 and A_2A_AR activation.

## 2. Materials and methods

### 2.1. Materials

PUE, TMP, strychnine (STR) and phenobarbital (PB) were purchased from the National Institute for the Control of Pharmaceutical and Biological Products (Beijing, China). Acetonitrile and formic acid (HPLC grade) were purchased from Merck KGaA (Darmstadt, Germany) and ROE scientific Inc. (Newark, USA). Borneol, α-asarone, selective A_1_ adenosine receptor antagonist (DPCPX), A_2A_ adenosine receptor antagonist (SCH58261) were purchased from Sigma-Aldrich (St. Louis, MO, USA). CD73 inhibitor adenosine 5′-( α, β-methylene) diphosphate sodium salt (APCP) was purchased from Bio-Techne China Co. Ltd (Shanghai, China). Dulbecco’s modified Eagle medium (DMEM), Fetal bovine serum (FBS), Penicillin-Streptomycin solution and Trypsin-EDTA solution were purchased from Gibco (Tulsa, OK, USA). Water was purified and demonized by Milli-Q ultrapure water purifications system.

### 2.2. Cell culture and conditions

The murine brain endothelial cells bEnd.3 cell lines were purchased from the American Type Culture Collection (Manassas, VA). The cells were cultured in DMEM medium supplemented with 10% (V/V) FBS and 1% (V/V) Penicillin-Streptomycin solution. The cells were incubated in a humidified atmosphere of 5% CO_2_ at 37 °C.

### 2.3. MTT assay

To evaluate the cytotoxicity of the drugs and to determine the optimal concentration of the drugs for *in vitro* experiments, the cell viability was measured by MTT assay. Briefly, bEnd.3 cells were seeded in a 96-well plates (5000 cells/well) and allowed to seed for 24 h, after which they were treated once with borneol (50, 100, 200 μM), α-asarone (50, 100, 200 μM), PUE (50, 100, 200 μM), TMP (50, 100, 200 μM), borneol (50, 100, 200 μM) with PUE (50 μM), α-asarone (50, 100, 200 μM) with PUE (50 μM), borneol (50, 100, 200 μM) with TMP (50 μM), α-asarone (50, 100, 200 μM) with TMP (50 μM) SCH58261 (25, 50, 100 μM), APCP (25, 50, 100 μM), DPCPX (25, 50, 100 μM). After each treatment (*n* = 6), the cells were incubated with 0.5 mg/ml MTT in DMEM for 4 h in dark condition and then carefully removed the supernatant and added 150 μL of DMSO under low speed shaking for 10 min to dissolve the formazan product. The optical density (OD) value of 490 nm was measured using the microplate reader (Thermo Fisher scientific, China).

### 2.4. *In vitro* BBB model

Monolayer bEnd.3 cells were used to simulate the blood–brain barrier(Fu, 2012). Briefly, cell culture inserts (Corning, NY, USA) were put into 12-well plates. Then, 5 × 10^4^ cells were seeded on each upper chamber with 500 μL 10% FBS DMEM medium, and the bottom chamber was filled with 1.5 mL of the medium. Then a trans-endothelial electrical resistance (TEER) instrument (EVOM2, WPI, USA) was used to measure the TEER. The TEER value was increasing with cell proliferation, and when the value became consistent (>200 Ω cm^2^) for seven consecutive days, the *in vitro* BBB was established (Yi et al., 2017; Zhu et al., 2018).

### 2.5. Effects of borneol and α-asarone on *in vitro* BBB permeability of PUE and TMP

The permeability of PUE and TMP across the *in vitro* BBB model with the action of borneol or α-asarone was evaluated. PUE or TMP (50 μmol/L) was added into the upper chamber, with borneol or α-asarone at a concentration of 50, 100 or 200 μmol/L respectively, and the controls were only PUE or only TMP (*n* = 3). After 24 h incubation, TEER values were measured to evaluate the integrity of monolayer bEnd.3 cells. Then, the medium in the bottom chamber was collected and analyzed by HPLC. The cumulative amount of PUE and TMP permeated the in vitro BBB model was calculated.

The supernatant of medium was collected after centrifugation at 15,000 rpm for 10 min and was analyzed by a validated HPLC method (Yang et al., 2016). The HPLC condition for PUE was as follow: Agilent TC-C18 (2) column (250 × 4.6 mm, 5 µm) maintained at temperature of 35 °C, the flow rate was 1.0 mL/min and the mobile phase was: (A) 65% aqueous phosphoric acid (0.1%) and (B) 30% methanol. The detection wavelength was set at λ = 296 nm. The HPLC condition for TMP was slightly different from PUE. The mobile phase was: (A) 60% aqueous phosphoric acid (0.1%) and (B) 40% methanol, and the detection wavelength was set at λ = 296 nm.

### 2.6. Effects on borneol and α-asarone on adenosine receptors

PUE or TMP (50 μmol/L) was added into the upper chamber, with borneol or α-asarone (200 μmol/L) respectively, and the controls received PUE or TMP only (*n* = 3). APCP, DPCPX, and SCH58261 (25 μmol/L) were used to inhibit CD73, A_1_AR or A_2A_AR respectively. After 24 h incubation, TEER value and amount of permeated PUE and TMP were measured as above described.

To analyze the protein levels in cell lysates after 24 h treatment with borneol or α-asarone, western blot analysis was performed. bEnd.3 cells were homogenized in 100–150 μL of RIPA lysis buffer containing 1% protease inhibitor and protein lysates were normalized using Bicinchoninic Acid (BCA) protein assay kit (Beyotime Biotechnology, Haimen, China). Protein lysates (40 μg) were loaded on to 10% SDS/PAGE gel separately, and then were transferred to polypropylene fluoride membranes, followed by blocking with 5% milk solution for 1 h. Primary antibody was incubated at 4 °C for overnight. After three times wash with TBST, the blots were incubated with second antibody for 2.5 hours at room temperature. After three times wash with TBST, the chemiluminescence signals of the membrane were detected by the EasySee Western Blot Kit (Beijing TransGen Biotech, Beijing, China). Image J software 1.43 (National Institutes of Health. Bethesda, MD) was used for densitometry analysis. Primary antibodies used were as follow: anti-ZO-1 (13663S, 1:1000, Cell Signaling Technology), anti-A_1_AR (ab82477, 1:1000, Abcam), anti-A_2A_AR (ab3461, 1:1000, Abcam) and anti-β-acting (4970S, 1:1000, Cell Signaling Technology). Second antibody: goat anti-rabbit IgG (ab97051, 1:5000, Abcam).

### 2.7. Pharmacokinetic study

Male Sprague-Dawley rats (220–260 g) were purchased from Changsha SLAC laboratory animal Co., Ltd (Changsha, China) and were housed in the Animal experiment center of Hunan Provincial People’s Hospital under room temperature (20–25 °C), with moderate humidity (50–60%) and 12 h day/night cycle. All animal experimental procedures were approved by the Animal Ethics Committee at the animal experiment center, Central South University. Rats were fasted for 12 h but were free to access water before experiments.

A total of 198 rats were randomly divided into three groups. Group 1 (*n* = 66) received PUE (20 mg/kg) and TMP (10 mg/kg), group 2 (*n* = 66) received PUE (20 mg/kg), TMP (10 mg/kg) and borneol (25 mg/kg), group 3 received PUE (20 mg/kg), TMP (10 mg/kg) and α-asarone (25 mg/kg). All agents were orally administrated. Plasma and cerebra samples were collected at 0.17, 0.33, 0.5, 0.75, 1, 1.5, 2, 3, 6, 8, 12 h (*n* = 6). The rats were sacrificed by excessive anesthesia, followed by quickly opening of the skull. After removing the whole brain and washing the blood on brain tissue with double distilled water, with drying using filter paper, right brain tissues (0.15 g), under dark conditions, were removed and then added into 1500 μL acetonitrile aqueous solution (60%, 4 °C). After homogenization, the magnetic beads were separated and the homogenate was collected. All the samples were stored at −80 °C before analysis.

### 2.8. LC-MS/MS analysis

The plasma sample or brain tissue homogenate (100 μL) was diluted by adding 300 μL of IS working solution (including PB and STR) and vortex-mixed for 5 min. After 15 min centrifugation at 10000 rpm, the supernatant was collected for analysis.

HPLC was performed on a XtimateTM C18 column (3.0 mm ×100 mm, 3-micro, Welch). Column temperature was maintained at 40 °C. The mobile phase was composed of A: 0.1% formic acid solution and B: acetonitrile. Gradient elution program was 0–1.5 min, 5% B; 11 min, 75% B; 13.5–15 min, 5% B. Total flow rate was 0.5 mL/min. The injection volume was 10 μL. Quantitation and monitoring of analysis were achieved on a 4000 QTRAP system (AB SCIEX, CA, USA) in the MRM mode using ESI with both negative and positive mode ionization. Mass parameters were as followed: spray gas 25 kPa; ion spray voltages 5000 V; temperature 600 °C; ion source GS1:70; ion source GS 2:70; collision-activated dissociation: high; interface heater: on. The validation of LC-MS/MS method are available in the Supplemental Data.

### 2.9. Statistical analyses

All data were shown as mean ± SD. Pharmacokinetics parameters were obtained using the DAS Version 3.3 software. Comparison between two groups was conducted using unpaired Student’s *t*-test. Comparison of multiple groups was conducted using one-way analysis of variance (ANOVA). Statistical significance was established at *p < .05, **p < .01.

## 3. Results and discussion

### 3.1. MTT assay

**Figure 1.**
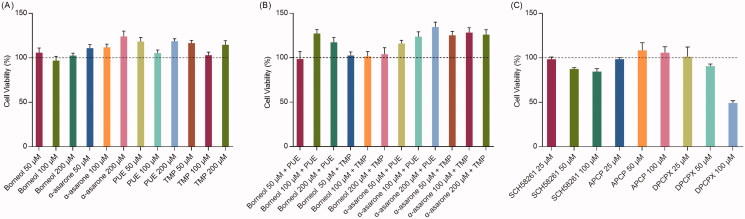
*In vitro* cytotoxicity assay (*n* = 6). (A) the effects of different concentration of drugs and adjuvant agents on cell viability; (B) the effects of combined use of drugs and adjuvant agents on cell viability, concentration for PUE and TMP were 50 μM; (C) the effects of different concentration of adenosine receptor inhibitors on cell viability.

Figure 1 shows the cell viability after treatment. Free borneol, α-asarone, PUE and TMP (50, 100, 200 μmol/L) all showed no cytotoxicity to bEnd.3 cells (Figure 1(A)). In contrast, free α-asarone, PUE, and TMP slightly increased cell proliferation. Also, when given combined, different concentration of borneol with PUE or TMP and different concentration of α-asarone with PUE or TMP showed no cytotoxicity (Figure 1(B)). Therefore, we chose the relatively lower concentration of 50 μmol/L, with no cytotoxicity, for further experiments.

As for inhibitors. APCP showed no cytotoxicity. However, both SCH58261 and DPCPX showed cytotoxicity at higher doses (50, 100 μmol/L) respectively (Figure 1(C)). Therefore, the concentration of 25 μmol/L was selected for further experiments.

### 3.2. Borneol and α-asarone increase *in vitro* BBB permeability of PUE and TMP by activating adenosine receptors

**Figure 2.**
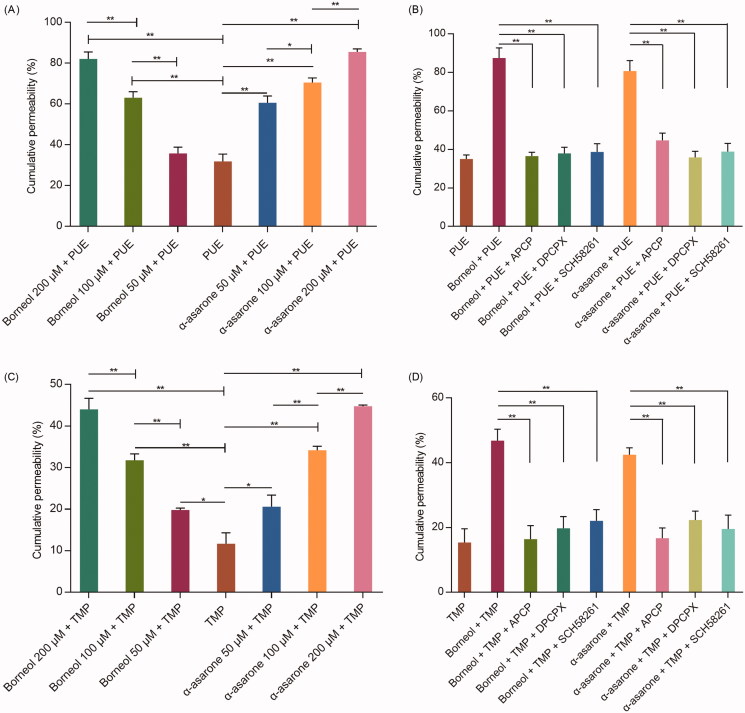
The cumulative permeability of PUE and TMP (*n* = 3). (A) effects of different concentration of borneol and α-asarone on the PUE cumulative permeability; (B) effects of adenosine receptor inhibitors on the PUE cumulative permeability; (C) effects of different concentration of borneol and α-asarone on the TMP cumulative permeability; (D) effects of adenosine receptor inhibitors on the TMP cumulative permeability. Concentration for PUE and TMP were 50 μM. *p < 0.05, **p < 0.01.

The effects of borneol and α-asarone on PUE and TMP permeability were shown in Figure 2. Both free PUE or TMP exhibited low cumulative permeability after 24 h incubation. When given combined with borneol or α-asarone, the cumulative permeability was significantly increased and a dose-response relationship was observed.

However, the increasing effects of borneol and α-asarone were counteracted when APCP, SCH58261 or DPCPX was added.

CD73 promotes the synthesis of adenosine, which activates A_1_AR and A_2A_AR and subsequently changes cytoskeleton and open paracellular junctions of BBB, thus increase the drug transport across the BBB. These results suggested that borneol or α-asarone could simultaneously activate A_1_AR and A_2A_AR, which were both important for drug permeation.

### 3.3. Western blot analysis

In CNS, vascular endothelial cells are tightly linked together by adhesive linker proteins, such as ZO-1 (zona occuldens-1), which severely restricts cell bypass transport to the brain. The structure and function of ZO-1 are closely correlated to other tight junction proteins, therefore, the disruption of ZO-1 would change the function of whole tight junctions (Tornavaca et al., 2015). ZO-1 is often used as an indicator for evaluation of integrity of the tight connection barriers of various tissues, including BBB, intestinal epithelial cells, corneal endothelial cells and cytoskeleton remodeling (Haseloff et al., 2015).

**Figure 3.**
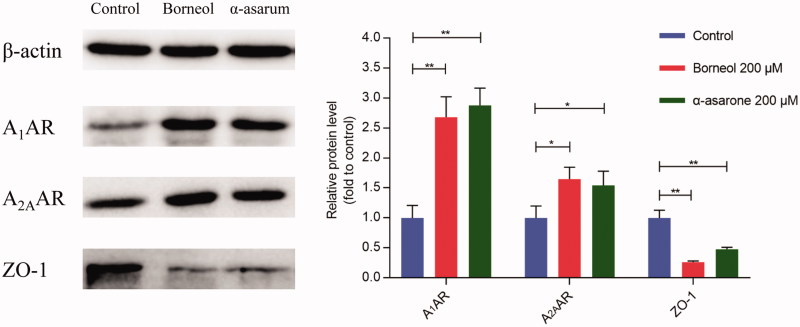
The protein expression of ZO-1, A1AR, and A2AR. Date are mean ± SEM (*n* = 3). *p < 0.05, **p < 0.01.

Western blotting results revealed that bEnd.3 cells that established BBB model highly expressed ZO-1 but not A_1_AR or A_2A_AR. After treatment of borneol or α-asarone (200 μmol/L) for 24 h, the expression of ZO-1 was significantly decreased and the expression A_1_AR and A_2A_AR were significantly increased (Figure 3). These results verified that both borneol or α-asarone could activate the AR signaling pathway and accordingly decrease the expression of tight junction protein ZO-1.

### 3.4. Pharmacokinetic study

**Figure 4.**
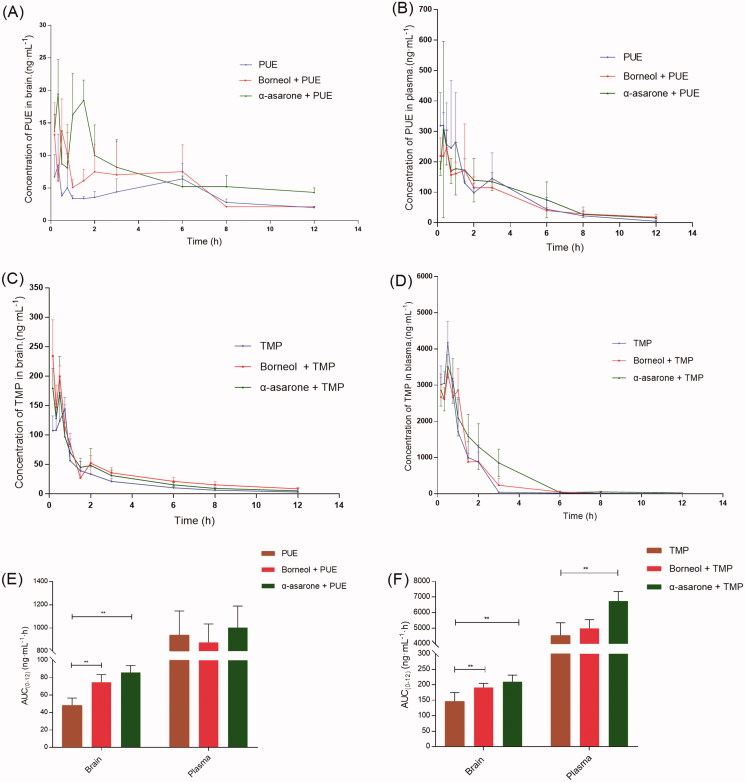
*In vivo* time–concentration profiles of PUE and TMP (*n* = 6). (A) the concentration–time profiles of PUE in brain tissue homogenate; (B) the concentration–time profiles of PUE in plasma; (C) the concentration–time profiles of TMP in brain tissue homogenate; (D) the concentration–time profiles of TMP in plasma; (E) comparison of PUE delivery into BBB (AUC brain) and in the plasma (AUC plasma); (F) comparison of TMP delivery into BBB (AUC brain) and in the plasma (AUC plasma). Doses orally administrate: PUE (20 mg/kg), TMP (10 mg/kg), Borneol (25 mg/kg) and α-asarone (25 mg/kg). *p < 0.05, **p < 0.01.

The brain concentration-time profile and plasma concentration-time profile of after oral administration of PUE (20 mg/kg) and TMP (10 mg/kg) without and with borneol (25 mg/kg) or α-asarone (25 mg/kg) were presented in Figure 4. The major pharmacokinetic parameters of PUE and TMP were summarized in Tables 1 and 2, respectively.

**Table 1.** Pharmacokinetic parameters of PUE in plasma and in brain tissue homogenate of rats.

Parameters	PUE (20 mg/kg)	PUE (20 mg/kg)+Borneol (25 mg/kg)	PUE (20 mg/kg)+α-asarone (25 mg/kg)
AUC_0-12 (Brain)_ (ng/mL h)	48.05 ± 8.54	64.80 ± 22.713**	86.02 ± 7.927**
MRT_0-12 (Brain)_ (h)	5.19 ± 0.22	4.39 ± 0.337*	4.64 ± 0.528*
t_1/2z (Brain)_ (h)	11.33 ± 4.07	5.62 ± 2.844*	12.81 ± 5.64
T_max (Brain)_ (h)	0.31 ± 0.13	0.54 ± 0.53	0.61 ± 0.52
C_max (Brain)_ (ng/mL)	13.24 ± 4.28	21.82 ± 9.33	35.16 ± 12.442**
AUC_0-12 (Plasma)_ (ng/mL h)	938.29 ± 208.57	874.95 ± 159.34	1003.32 ± 185.60
MRT_0-12 (Plasma)_ (h)	2.81 ± 0.78	3.36 ± 0.39	3.42 ± 0.19
t_1/2z (Plasma)_ (h)	1.66 ± 0.59	4.31 ± 1.991**	3.39 ± 0.824*
T_max (Plasma)_ (h)	0.38 ± 0.22	0.56 ± 0.49	0.85 ± 0.61
C_max (Plasma)_ (ng/mL)	439.15 ± 137.02	367.99 ± 201.93	368.58 ± 260.71

*p < .05 compared with the control group PUE without borneol or α-asarone.**p < .01 compared with the control group PUE without borneol or α-asarone.

**Table 2.** Pharmacokinetic parameters of TMP in plasma and in brain tissue homogenate of rats.

Parameters	TMP (10 mg/kg)	TMP (10 mg/kg)+Borneol (25 mg/kg)	TMP (10 mg/kg)+α-asarone (25 mg/kg)
AUC_0-12 (Brain)_ (ng/mL h)	146.86 ± 27.90	191.42 ± 13.334**	210.28 ± 21.425**
MRT_0-12 (Brain)_ (h)	0.78 ± 0.06	0.70 ± 0.042*	1.05 ± 0.099**
t_1/2z (Brain)_ (h)	0.68 ± 0.39	0.48 ± 0.28	0.81 ± 0.26
T_max (Brain)_ (h)	0.63 ± 0.14	0.33 ± 0.183**	0.28 ± 0.172**
C_max (Brain)_ (ng/mL)	153.18 ± 18.24	243.35 ± 51.477**	203.65 ± 39.538*
AUC_0-12 (Plasma)_ (ng/mL h)	4532.32 ± 819.20	4990.68 ± 559.46	6739.15 ± 615.382**
MRT_0-12 (Plasma)_ (h)	0.99 ± 0.14	1.23 ± 0.141*	1.61 ± 0.225**
t_1/2z (Plasma)_ (h)	1.24 ± 0.73	0.96 ± 0.25	1.00 ± 0.36
T_max (Plasma)_ (h)	0.50 ± 0.00	0.61 ± 0.33	0.51 ± 0.33
C_max (Plasma)_ (ng/mL)	4171.39 ± 585.80	3377.59 ± 189.79	3989.03 ± 985.88

*p < .05 compared with the control group TMP without borneol or α-asarone.**p < .01 compared with the control group TMP without borneol or α-asarone.

Co-administration of borneol or α-asarone enhanced brain distribution of PUE. 1.34-fold and 1.79-fold increase in AUC_0-12 (Brain)_ of PUE were observed when combined administrated with borneol or α-asarone. However, the AUC_0-12 (plasma)_ of PUE did not change significantly. Co-administration of borneol or α-asarone also enhanced brain distribution of TMP. 1.30-fold and 1.43-fold increase in AUC_0-12 (Brain)_ of TMP were observed when combined administrated with borneol or α-asarone. Borneol did not alter the AUC_0-12 (plasma)_ of PUE, but α-asarone significantly enhanced AUC_0-12 (plasma)_ of PUE by 1.48-fold.

These results indicated that oral administration of PUE and TMP combined with borneol or α-asarone had an increased brain distribution. The previous study reported that borneol not only increased brain distribution but also increased oral absorption of PUE, but this study reported only enhanced brain distribution (Yi et al., 2017). This may be because the doses of PUE (20 mg/kg) and borneol (25 mg/kg) used in our study were relatively lower than the previous study (PUE: 200 mg/kg, borneol: 50, 100 and 200 mg/kg). But it suggested that low dose of borneol could also enhance brain distribution of PUE and TMP.

While borneol and α-asarone are both aromatic resuscitation drugs, the increasing effects of α-asarone on PUE and TMP across the BB was firstly reported. Moreover, α-asarone exhibited superior “orifice-opening” effects on BBB even at low dose, and higher AUC_0-12 (Brain)_ of PUE and TMP, as well as higher AUC0-12_(plasma)_ of PUE and TMP, were observed.

Several mechanisms of borneol increasing brain distribution of PUE have been proposed, such as inhibition of P-glycoprotein (P-gp), enhancement of transmembrane tight junction protein and enhancement of vasodilatory neurotransmitters (Zhang et al., 2017). Here, we reported that the permeability-enhancing effects of borneol and α-asarone may also be associated with activation of adenosine receptors. Borneol and α-asarone are both potent ‘orifice-opening’ agents and could be used as adjuvant agents for brain delivery.

## 4. Conclusion

In this study, the effects of borneol and α-asarone as agents for increasing BBB permeability of PUE and TMP were investigated. Low dose of borneol did not improve oral bioavailability of PUE and TMP, but increased BBB permeability; low dose of α-asarone not only improved oral bioavailability of PUE and TMP but also increased BBB permeability, and the enhancing effects might be better than borneol. The underlying mechanism for the enhancing effects of borneol and α-asarone on BBB permeability may be associated with activation of adenosine receptors. Both borneol and α-asarone are promising adjuvant agents for increasing oral delivery of PUE and TMP to brain.

## Supplementary Material

Supplemental Material
