# Supplementary material for: Borneol and Α-asarone as adjuvant agents for improving blood–brain barrier permeability of puerarin and tetramethylpyrazine by activating adenosine receptors
Source: Drug Deliv. 2018 Oct 19;25(1):1858–64. doi: 10.1080/10717544.2018.1516005 (PMC6201807; doi:10.1080/10717544.2018.1516005)
Supplement: Supplemental Material [file IDRD_A_1516005_SM9663.docx]

**Supplementary information**

**Borneol and α-asarone as adjuvant agents for improving blood-brain barrier permeability of puerarin and tetramethylpyrazine by activating adenosine receptors**

Jun-Yong Wu^1,2,3^, Yong-Jiang Li^1,2,3^, Le-Yang^1,2,3^, Yi-Yun Hu^1,2,3^, Xiong-Bin Hu^1,2,3^, Tian-Tian Tang^1,2,3^, Jie-Min Wang^1,2,3^, Xin-Yi Liu^1,2,3^, Da-Xiong Xiang^1,2,3,*^

1. Department of Pharmacy, The Second Xiangya Hospital, Central South University, Changsha 410011, Hunan, China
2. Institute of Clinical Pharmacy, Central South University, Changsha 410011, Hunan, China
3. Institute of Clinical Pharmacy, Central South University, Changsha 410011, Hunan, China

* Corresponding author at:

Department of Pharmacy, The Second Xiangya Hospital, Central South University, Changsha 410011, Hunan, China; Email: xiangdaxiong@csu.edu.cn

A


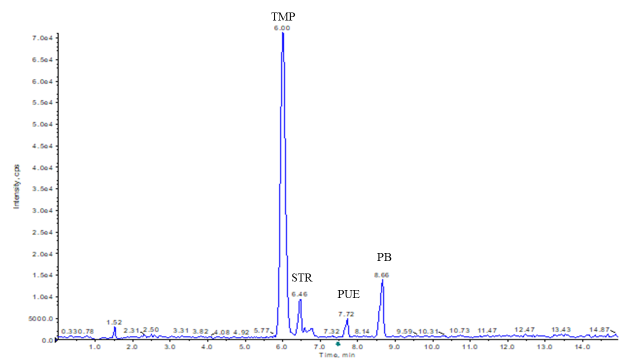


B


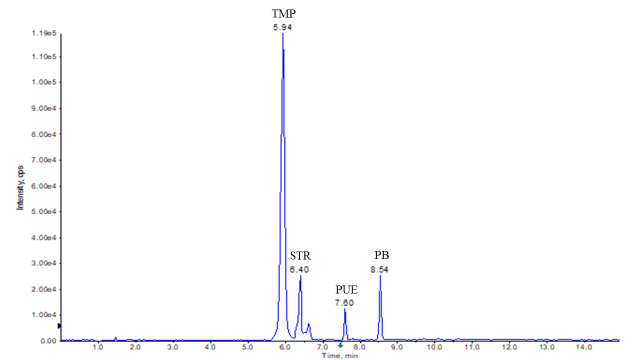


Figure S1. (A)The chromatogram map of mixed standard plasma sample. (B)The chromatogram map of mixed standard brain tissue homogenate sample. PB: Phenobarbital; STR: strychnine; TMP: Tetramethylphrazine; PUE: Puerarin


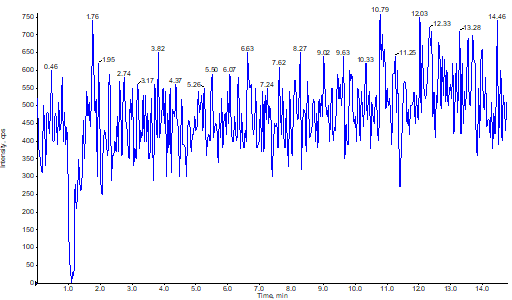


A

B


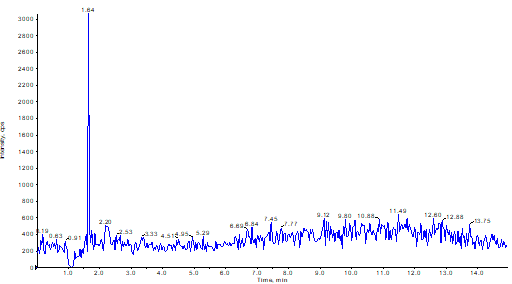


Figure S2. (A) The chromatogram map of blank plasma sample; (B) The chromatogram map of blank brain tissue homogenate sample.

Table S1. Linear regression equations, correlation coefficients and the limit of quantitation for PUE and TMP in plasma samples.

|  | Linear range  (ng mL^-1^) | Linear regression equations | correlation coefficients (R^2^) | limit of quantitation (ng mL^-1^) |
| --- | --- | --- | --- | --- |
| PUE | 1.17 — 1000 | y = 0.0188x - 0.0167 | 0.9999 | 0.58 |
| TMP | 8.67 — 1833 | y = 0.0049x + 0.0871 | 0.9987 | 6.51 |

Table S2. Linear regression equations, correlation coefficients and the limit of quantitation for PUE and TMP in brain tissue homogenate samples.

|  | Linear range  (ng mL^-1^) | Linear regression equations | correlation coefficients (R^2^) | limit of quantitation (ng mL^-1^) |
| --- | --- | --- | --- | --- |
| PUE | 1.17 — 1000 | y = 0.0101x - 0.0114 | 0.9999 | 0.45 |
| TMP | 8.67 — 1833 | y = 0.0087x + 0.0480 | 0.9998 | 5.33 |

Table S3. Matrix effects of PUE in plasma samples.

|  | Low dose (2.5ng mL^-1^) | | Median dose (66.7ng mL^-1^) | | High dose (800ng mL^-1^) | |
| --- | --- | --- | --- | --- | --- | --- |
|  | A_1_ | A_0_ | A_1_ | A_0_ | A_1_ | A_0_ |
| Area | 3569 | 3800 | 156428 | 142529 | 1589854 | 1829686 |
|  | 3346 | 4069 | 153926 | 151997 | 1646856 | 1766822 |
|  | 2946 | 4057 | 151882 | 139639 | 1658973 | 1596833 |
|  | 3128 | 3370 | 161367 | 144377 | 1766364 | 1878782 |
|  | 3558 | 3823 | 152991 | 143933 | 1807060 | 1795511 |
| RSD (%) | 8.21 | 7.41 | 2.43 | 3.17 | 5.31 | 6.05 |
| Matrix effects (%) | 86.55 | | 107.49 | | 95.51 | |

Table S4. Matrix effects of TMP in plasma samples.

|  | Low dose (16.7ng/mL) | | Median dose (200ng/mL) | | High dose (1500ng/mL) | |
| --- | --- | --- | --- | --- | --- | --- |
|  | A_1_ | A_0_ | A_1_ | A_0_ | A_1_ | A_0_ |
| Area | 21401 | 25074 | 297580 | 315496 | 2021970 | 1931426 |
|  | 25511 | 24846 | 298730 | 324566 | 1858499 | 2024699 |
|  | 26550 | 25467 | 283502 | 309271 | 1935335 | 1886077 |
|  | 28350 | 27391 | 297915 | 307196 | 1810692 | 1879648 |
|  | 24355 | 26631 | 313430 | 308591 | 1969334 | 2065324 |
| RSD (%) | 10.29 | 4.20 | 3.55 | 2.30 | 4.42 | 4.27 |
| Matrix effects (%) | 97.49 | | 95.27 | | 98.04 | |

Table S5. Matrix effects of PUE in brain tissue homogenate samples.

|  | Low dose (2.5ng mL^-1^) | | Median dose (66.7ng mL^-1^) | | High dose (800ng mL^-1^) | |
| --- | --- | --- | --- | --- | --- | --- |
|  | A_1_ | A_0_ | A_1_ | A_0_ | A_1_ | A_0_ |
| Area | 1826 | 2256 | 82512 | 88479 | 826294 | 784904 |
|  | 2096 | 2159 | 76012 | 86151 | 723373 | 780847 |
|  | 1930 | 2521 | 79188 | 86415 | 719407 | 899115 |
|  | 1926 | 2265 | 82469 | 85741 | 791131 | 825247 |
|  | 2102 | 2312 | 90216 | 91256 | 746065 | 766638 |
| RSD (%) | 6.06 | 5.83 | 6.44 | 2.62 | 6.07 | 6.62 |
| Matrix effects (%) | 85.82 | | 93.69 | | 93.83 | |

Table S6. Matrix effects of TMP in brain tissue homogenate samples.

|  | Low dose (16.7ng/mL) | | Median dose (200ng/mL) | | High dose (1500ng/mL) | |
| --- | --- | --- | --- | --- | --- | --- |
|  | A_1_ | A_0_ | A_1_ | A_0_ | A_1_ | A_0_ |
| Area | 16849 | 16616 | 194056 | 200590 | 2043952 | 1973054 |
|  | 16055 | 15113 | 195611 | 209741 | 1947170 | 2035847 |
|  | 15187 | 17420 | 193399 | 205508 | 1951649 | 1927725 |
|  | 16976 | 13961 | 201656 | 211731 | 2100273 | 1896512 |
|  | 16304 | 14394 | 208621 | 200936 | 2022783 | 2061531 |
| RSD (%) | 4.40 | 9.50 | 3.25 | 2.45 | 3.22 | 3.53 |
| Matrix effects (%) | 104.99 | | 96.58 | | 101.73 | |

Table S7. Accuracy, intra-day and inter-day recovery of PUE in plasma samples.

|  | Low dose  (2.5ng mL^-1^) | | Median dose (66.7ng mL^-1^) | | High dose  (800ng mL^-1^) | |
| --- | --- | --- | --- | --- | --- | --- |
|  | Concentration (μg/ml) | Recovery (%) | Concentration (μg/ml) | Recovery (%) | Concentration (μg/ml) | Recovery (%) |
| Day 1 | 2.49 | 99.46 | 68.71 | 103.02 | 1096.76 | 109.68 |
|  | 2.47 | 98.72 | 68.27 | 102.36 | 1079.28 | 107.93 |
|  | 2.55 | 101.89 | 61.61 | 92.37 | 1063.29 | 106.33 |
|  | 2.48 | 99.29 | 58.51 | 87.72 | 977.74 | 97.77 |
|  | 2.34 | 93.50 | 62.66 | 93.95 | 1065.65 | 106.57 |
| Day 2 | 2.55 | 102.15 | 68.27 | 102.36 | 1060.05 | 106.00 |
|  | 2.72 | 108.75 | 61.61 | 92.37 | 1064.08 | 106.41 |
|  | 2.43 | 97.37 | 58.51 | 87.72 | 1073.99 | 107.40 |
|  | 2.13 | 85.15 | 62.66 | 93.95 | 1002.76 | 100.28 |
|  | 2.22 | 88.73 | 72.91 | 109.32 | 968.74 | 96.87 |
| Day 3 | 2.36 | 94.42 | 66.94 | 100.36 | 968.95 | 96.90 |
|  | 2.28 | 91.31 | 69.62 | 104.38 | 892.65 | 89.26 |
|  | 2.63 | 105.20 | 68.55 | 102.78 | 956.81 | 95.68 |
|  | 2.33 | 93.32 | 67.90 | 101.80 | 1001.05 | 100.11 |
|  | 2.66 | 106.31 | 68.12 | 102.13 | 1047.63 | 104.76 |
| RSD_intra-day_ (%) | 3.13 | | 6.91 | | 4.36 | |
| RSD_inter-day_ (%) | 3.52 | | 6.74 | | 3.87 | |

Table S8. Accuracy, intra-day and inter-day recovery of TMP in plasma samples.

|  | Low dose (16.7ng/mL) | | Median dose (200ng/mL) | | High dose (1500ng/mL) | |
| --- | --- | --- | --- | --- | --- | --- |
|  | Concentration (μg/ml) | Recovery (%) | Concentration (μg/ml) | Recovery (%) | Concentration (μg/ml) | Recovery (%) |
| Day 1 | 16.71 | 100.06 | 220.31 | 110.16 | 1489.21 | 99.28 |
|  | 16.93 | 101.38 | 201.42 | 100.71 | 1429.94 | 95.33 |
|  | 15.88 | 95.09 | 206.15 | 103.08 | 1592.24 | 106.15 |
|  | 14.78 | 88.50 | 195.84 | 97.92 | 1538.40 | 102.56 |
|  | 16.49 | 98.74 | 197.68 | 98.84 | 1682.14 | 112.14 |
| Day 2 | 16.31 | 97.66 | 205.54 | 102.77 | 1456.78 | 97.12 |
|  | 16.99 | 101.74 | 199.14 | 99.57 | 1435.20 | 95.68 |
|  | 17.31 | 103.65 | 203.23 | 101.62 | 1459.29 | 97.29 |
|  | 16.54 | 99.04 | 191.25 | 95.63 | 1589.14 | 105.94 |
|  | 17.34 | 103.83 | 195.42 | 97.71 | 1572.84 | 104.86 |
| Day 3 | 15.98 | 95.69 | 192.54 | 96.27 | 1529.27 | 101.95 |
|  | 16.91 | 101.26 | 198.09 | 99.05 | 1548.75 | 103.25 |
|  | 15.84 | 94.85 | 199.08 | 99.54 | 1548.19 | 103.21 |
|  | 16.59 | 99.34 | 209.17 | 104.59 | 1620.20 | 108.01 |
|  | 15.92 | 95.33 | 203.85 | 101.93 | 1438.20 | 95.88 |
| RSD_intra-day_ (%) | 5.35 | | 4.79 | | 6.26 | |
| RSD_inter-day_ (%) | 5.08 | | 2.28 | | 6.65 | |

Table S9. Accuracy, intra-day and inter-day recovery of PUE in brain tissue homogenate samples.

|  | Low dose  (2.5ng mL^-1^) | | Median dose (66.7ng mL^-1^) | | High dose  (800ng mL^-1^) | |
| --- | --- | --- | --- | --- | --- | --- |
|  | Concentration (μg/ml) | Recovery (%) | Concentration (μg/ml) | Recovery (%) | Concentration (μg/ml) | Recovery (%) |
| Day 1 | 2.59 | 103.54 | 60.83 | 91.21 | 829.95 | 99.60 |
|  | 2.46 | 98.27 | 69.48 | 104.17 | 814.83 | 97.78 |
|  | 2.59 | 103.49 | 74.21 | 111.27 | 853.93 | 102.48 |
|  | 2.69 | 107.61 | 58.23 | 87.31 | 839.93 | 100.80 |
|  | 2.42 | 96.94 | 66.50 | 99.70 | 807.19 | 96.87 |
| Day 2 | 2.73 | 109.35 | 69.23 | 103.80 | 815.39 | 97.85 |
|  | 2.46 | 98.36 | 63.39 | 95.04 | 814.93 | 97.80 |
|  | 2.44 | 97.41 | 55.50 | 83.20 | 845.35 | 101.45 |
|  | 2.55 | 102.12 | 69.49 | 104.19 | 835.24 | 100.23 |
|  | 2.82 | 112.94 | 70.34 | 105.46 | 839.48 | 100.74 |
| Day 3 | 2.58 | 103.17 | 81.49 | 122.18 | 831.29 | 99.76 |
|  | 2.14 | 85.70 | 72.50 | 108.69 | 815.48 | 97.86 |
|  | 2.10 | 83.95 | 69.49 | 104.19 | 825.23 | 99.03 |
|  | 2.46 | 98.32 | 59.29 | 88.89 | 856.95 | 102.84 |
|  | 2.48 | 99.18 | 64.23 | 96.30 | 855.04 | 102.61 |
| RSD_intra-day_ (%) | 4.26 | | 9.80 | | 2.27 | |
| RSD_inter-day_ (%) | 5.33 | | 8.72 | | 2.39 | |

Table S10. Accuracy, intra-day and inter-day recovery of TMP in brain tissue homogenate samples.

|  | Low dose (16.7ng/mL) | | Median dose (200ng/mL) | | High dose (1500ng/mL) | |
| --- | --- | --- | --- | --- | --- | --- |
|  | Concentration (μg/ml) | Recovery (%) | Concentration (μg/ml) | Recovery (%) | Concentration (μg/ml) | Recovery (%) |
| Day 1 | 15.60 | 93.44 | 184.59 | 92.29 | 1432.95 | 95.53 |
|  | 17.63 | 105.57 | 182.96 | 91.48 | 1643.20 | 109.55 |
|  | 16.29 | 97.57 | 194.46 | 97.23 | 1390.44 | 92.70 |
|  | 16.84 | 100.85 | 199.48 | 99.74 | 1523.13 | 101.54 |
|  | 15.90 | 95.23 | 205.18 | 102.59 | 1487.39 | 99.16 |
| Day 2 | 15.82 | 94.75 | 220.48 | 110.24 | 1429.39 | 95.29 |
|  | 16.53 | 99.01 | 217.29 | 108.65 | 1565.04 | 104.34 |
|  | 16.83 | 100.79 | 198.48 | 99.24 | 1590.34 | 106.02 |
|  | 17.65 | 105.72 | 204.41 | 102.20 | 1580.76 | 105.38 |
|  | 17.43 | 104.39 | 203.29 | 101.65 | 1589.02 | 105.93 |
| Day 3 | 16.85 | 100.92 | 185.93 | 92.96 | 1459.49 | 97.30 |
|  | 15.36 | 91.96 | 204.29 | 102.15 | 1529.29 | 101.95 |
|  | 17.88 | 107.05 | 192.40 | 96.20 | 1603.02 | 106.87 |
|  | 17.99 | 107.71 | 198.23 | 99.12 | 1521.25 | 101.42 |
|  | 15.22 | 91.16 | 201.43 | 100.72 | 1486.39 | 99.09 |
| RSD_intra-day_ (%) | 4.89 | | 4.93 | | 6.48 | |
| RSD_inter-day_ (%) | 4.55 | | 6.90 | | 6.53 | |
